# Elevated cyclin B2 expression in invasive breast carcinoma is associated with unfavorable clinical outcome

**DOI:** 10.1186/1471-2407-13-1

**Published:** 2013-01-02

**Authors:** Emman Shubbar, Anikó Kovács, Shahin Hajizadeh, Toshima Z Parris, Szilárd Nemes, Katrin Gunnarsdóttir, Zakaria Einbeigi, Per Karlsson, Khalil Helou

**Affiliations:** 1 Sahlgrenska Cancer Center, Department of Clinical Genetics, Institute of Biomedicine, Sahlgrenska Academy at University of Gothenburg, Gothenburg SE-41345, Sweden; 2 Pathology section, Department of Pathology, Sahlgrenska University Hospital, Gothenburg, SE-41345, Sweden; 3 Sahlgrenska Cancer Center, Department of Oncology, Institute of Clinical Sciences, Sahlgrenska Academy at University of Gothenburg, Gothenburg, SE-41345, Sweden; 4 Regional Cancer Centre (West), Western Sweden Health Care Region, Sahlgrenska University Hospital, Gothenburg, SE-41345, Sweden

**Keywords:** CCNB2, Invasive breast carcinoma, Prognostic marker

## Abstract

**Background:**

Breast cancer is a potentially fatal malignancy in females despite the improvement in therapeutic techniques. The identification of novel molecular signatures is needed for earlier detection, monitoring effects of treatment, and predicting prognosis. We have previously used microarray analysis to identify differentially expressed genes in aggressive breast tumors. The purpose of the present study was to investigate the prognostic value of the candidate biomarkers *CCNB2*, *ASPM*, *CDCA7*, *KIAA0101,* and *SLC27A2* in breast cancer.

**Methods:**

The expression levels and subcellular localization of the CCNB2, ASPM, CDCA7, KIAA0101, and SLC27A2 proteins were measured using immunohistochemistry (IHC) on a panel of 80 primary invasive breast tumors. Furthermore, the mRNA levels of *CCNB2*, *KIAA0101,* and *SLC27A2* were subsequently examined by qRT-PCR to validate IHC results. Patient disease-specific survival (DSS) was evaluated in correlation to protein levels using the Kaplan-Meier method. Multivariate Cox regression analysis was used to determine the impact of aberrant protein expression of the candidate biomarkers on patient DSS and to estimate the hazard ratio at 8-year follow-up.

**Results:**

Elevated cytoplasmic CCNB2 protein levels were strongly associated with short-term disease-specific survival of breast cancer patients (≤ 8 years; *P*<0.001) and with histological tumor type (*P*= 0.04). However, no association with other clinicopathological parameters was observed. Multivariate Cox regression analysis specified that CCNB2 protein expression is an independent prognostic marker of DSS in breast cancer. The predictive ability of several classical clinicopathological parameters was improved when used in conjunction with CCNB2 protein expression (C-index = 0.795) in comparison with a model without CCNB2 expression (C-index = 0.698). The protein levels of ASPM, CDCA7, KIAA0101, and SLC27A2 did not correlate with any clinicopathological parameter and had no influence on DSS. However, a significant correlation between the expression of the CCNB2 and ASPM proteins was detected (*P* = 0.03).

**Conclusion:**

These findings suggest that cytoplasmic CCNB2 may function as an oncogene and could serve as a potential biomarker of unfavorable prognosis over short-term follow-up in breast cancer.

## Background

Worldwide, breast cancer is responsible for more than half a million deaths each year, but the ability to predict clinical outcome of the disease is still limited [1]. Breast cancer is a complex multi-gene disease involving the activation of oncogenes, loss of tumor suppressor genes, and disruption of vital cell-signaling pathways responsible for cell survival, growth, differentiation, and apoptosis. In recent years, the cellular and molecular characterization of breast cancer has catalyzed a shift toward the development of improved diagnosis and treatment of this disease [2,3]. To improve long-term survival rates and quality of life, several treatment regimens are currently available, including surgery combined with adjuvant therapy. Furthermore, several clinicopathological factors are also used to stratify patients into groups with different prognoses and to predict their response to adjuvant systemic therapies, including histological tumor grade, stage, size, age at diagnosis, axillary lymph node status, human epidermal growth factor receptor 2 (HER2/*neu)* status, steroid hormone receptor expression, and vascular invasion [4,5]. However, despite improvements in the treatment of breast cancer, it remains the second most common cause of death in women after lung cancer. Moreover, breast cancer incidence has risen steadily in recent years and many patients are exposed to ineffective therapies, as well as, to unnecessary treatment-related toxicity [6]. Therefore, there is intense focus on the development of improved treatment for breast cancer, especially targeted therapies.

Recently, we performed a detailed analysis of copy number and gene expression in 97 primary invasive diploid breast tumors [7]. We identified molecular gene signatures in aggressive tumors that resulted in different clinical outcomes. In the present investigation, five genes (*CCNB2, CDCA7, ASPM, KIAA0101*, and *SLC27A2*) were selected from these gene signatures based on their significantly deregulated gene expression according to short-term disease-specific survival, triple-negative status, and/or stratified according to histological grade as defined by Bloom, Richardson, Elston/Ellis (BRE) grading system [8]. In addition, *CCNB2, ASPM, KIAA0101*, and *CDCA7* are known to be involved in DNA repair, DNA replication, and cell cycle arrest [9-13], whereas *SLC27A2* plays a role in fatty acid transport [14]. We then further analyzed their protein levels and subcellular localization in relation to patient clinical outcome as well as with clinicopathological features in an independent cohort of 80 primary invasive breast tumors.

## Methods

### Tumor specimens

Primary invasive tumors were obtained from 80 patients who had undergone surgery from the 1990 to 2006 at Sahlgrenska University Hospital, Gothenburg, Sweden. Formalin-fixed, paraffin-embedded tissues (FFPE) and fresh-frozen primary invasive breast carcinomas were obtained from the Departments of Pathology and Oncology at Sahlgrenska University Hospital in accordance with the Declaration of Helsinki and approved by the Medical Faculty Research Ethics Committee (Gothenburg, Sweden). The clinical and morphologic characteristics of the tumors are summarized in Table 1. To examine potential variations in protein expression of candidate biomarkers, the tumors were stratified according to disease-specific survival (DSS) with 8-year censoring, and histological grade as defined by Bloom, Richardson, Elston/Ellis (BRE) grading system [8].

**Table 1:** Clinicopathological characteristics of 80 invasive breast cancer patients

**Characteristics**	**pBRE I-II Survivors ≥8years (n = 21)**	**pBRE III Survivors ≥8years (n = 19)**	**pBRE I-II Survivors < 8 years (n = 20)**	**pBRE III Survivors <8 years (n = 20)**
**Mean age (y)**	58 (39–72)	52 (27–7)	55 (39–71)	53 (33–72)
**Histologic type**				
Ductal	15 (71)	18 (95)	15 (75)	16 (80)
Lobular	2 (10)	1 (5)	4 (20)	3 (15)
Ductal + lobular	0 (0)	0 (0)	1 (5)	0 (0)
Other	3 (14)	0 (0)	0 (0)	1 (5)
Not available	1 (5)	0 (0)	0 (0)	0 (0)
**Pathologic tumor size (mm)**				
pT1 (0–20)	2 (9)	6 (32)	3 (15)	5 (25)
pT2 (>20-50)	18 (86)	9 (47)	16 (80)	13 (65)
pT3 (>50)	1 (5)	4 (21)	1 (5)	2 (10)
pT4	0 (0)	0 (0)	0 (0)	0 (0)
**BRE grade**				
I-II	21 (100)		20 (100)	
III		19 (100)		20 (100)
**No. of axillary lymph nodes**				
0	7 (33)	7 (37)	7 (35)	7 (35)
1-3	5 (24)	7 (37)	6 (30)	6 (30)
≥4	9 (43)	5 (26)	7 (35)	7 (35)
**Surgery**				
Lumpectomy	9 (43)	6 (32)	9 (45)	6 (30)
Mastectomy	12 (57)	13 (68)	11 (55)	14 (70)
**ER/PR status**				
Negative	11 (52)	8 (42)	13 (65)	7 (35)
Positive	9 (43)	11 (58)	7 (35)	13 (65)
Not available	1 (5)			
**HER2/*neu *status**				
Positive	7 (33)	8 (42)	8 (40)	3 (15)
Negative	11 (52)	10 (53)	11 (55)	9 (45)
Not available	3 (15)	1 (5)	1 (5)	8 (40)

(N) = No. of patients (%).

BRE, Bloom, Richardson, Elston/Ellis; ER/PR: Estrogen/progesterone receptor.

### Immunohistochemistry (IHC)

Antibodies corresponding to CCNB2, CDCA7, KIAA0101, SLC27A2, and ASPM were optimized using 12 independent primary invasive breast tumors with different stage I-III as controls. Four micrometer FFPE sections were applied onto positively charged slides (FLEX IHC microscope slides, Dako, Sweden) and subsequently immunostained with rabbit anti-CCNB2 (Sigma-Aldrich, Stockholm, Sweden, HPA008873, 1:100 dilution), rabbit anti-CDCA7 (Sigma-Aldrich, HPA005565, 1:50 dilution), rabbit anti-SLC27A2 (Sigma-Aldrich, HPA026089, 1:50 dilution), mouse anti-KIAA0101 (Abnova, Stockholm, Sweden, H00009768-M01, 1:200 dilution), and rabbit anti-ASPM (Novus Biologicals, England, UK, 25970002, 1:1100 dilution) to determine protein expression levels and subcellular localization of the corresponding proteins in breast tumors. The FFPE sections were processed with the Dako EnVision™ FLEX antigen retrieval EDTA buffer (pH 9) for 20 minutes at 97°C using DAKO PT Link module (PT Link, Dakocytomation, Denmark) according to the manufacturer's instructions. The IHC procedure was performed using DAKO stainer (DAKO Auotstainer plus, Dakocytomation, Denmark) following the manufacturer's instructions. Antibody staining was evaluated by a single pathologist (AK). At the time of examination, the pathologist was blinded as to the diagnosis and other clinicopathological data. Immunoreactivity was defined as negative with a score of 0 (no staining in any cells or very weak cytoplasmic or nucleus staining in less than 10% of the invasive tumor cells). Positive immunoreactivity was defined as 1+ (weak to moderate staining in more than 10% of the invasive tumor cells) or 2+ (moderate to strong staining in more than 10% of the invasive tumor cells). Areas with intraductal carcinoma were excluded from the evaluation.

### Fluorescence *in situ* hybridization (FISH)

To assess *HER2/neu* gene status in the 67/80 available fresh-frozen tumor samples, fluorescence *in situ* hybridization was performed. A bacterial artificial chromosome (BAC) clone covering the *HER2/neu* locus (RP11-94L15) was purchased from BACPAC Resource Center (Oakland, CA, USA, http://bacpac.chori.org/) and used as a FISH probe. FISH was performed on tumor touch-prints prepared from fresh-frozen tumors as described elsewhere [15]. The analysis was performed on a Leica DMRA2 fluorescence microscope (Leica, Stockholm, Sweden) equipped with an ORCA Hamamatsu charged-couple devices camera (Hamamatsu Corporation, Stockholm, Sweden). Scoring of HER2/*neu* hybridization signals was carried out in each tumor specimen by counting the number of signals in at least 100 nuclei. Specimens were scored as either positive (1) when *HER2/neu* gene amplifications were detected in more than 10% of the analyzed cells or negative (0) in all other cases.

### Quantitative real-time PCR (qRT-PCR)

Total RNA was isolated from fresh-frozen tumor specimens using TRIzol reagent (Life Technologies, Stockholm, Sweden) and the Qiagen RNeasy mini kit (Qiagen, Stockholm, Sweden) according to the manufacturer's instructions, followed by treatment with RNase-free DNase (Ambion, Texas, USA). One microgram total RNA was converted to cDNA using random hexamers and Superscript III (Life Technologies) according to standard procedures. Validation of the IHC and FISH results was performed using qRT-PCR with TaqMan Gene Expression Assays (Life Technologies) for *CCNB2* (Hs00270424_m1), *KIAA0101* (Hs00207134_m1), *SLC27A2* (Hs00186324_m1), and HER2/*neu* (Hs01001580_m1) on a cohort of 62/80 tumors which were also used in the IHC and FISH analyses. The qRT-PCR reactions for each sample were performed in duplicate in independent experiments.

The *HPRT1* gene (Hs02800695_m1) was initially selected as an endogenous control because it exhibited low variance in mRNA expression between samples (data not shown). The qRT-PCR reactions (10 μl total) included 2 μl of cDNA template, 2x TaqMan Universal PCR Master Mix (ABI, Foster City, USA), and 1x FAM labeled gene-specific assay. All qRT-PCR reactions were performed in 384-well plates using the ABI PRISM 7900HT Sequence Detection System (ABI, Foster City, USA) with an initiation step at 95°C for 10 minutes, followed by 40 cycles at 95°C for 15 seconds and at 60°C for 1 minute. For each assay, a template dilution standard curve (5-fold range) was recorded. Genomic DNA and no-template samples were included as controls. Relative gene expression levels were calculated with the relative standard curve method using CT values of the analyzed genes normalized with *HPRT1*[16].

### Statistical analysis

Protein expression was examined in relation to DSS using the Kaplan-Meier method and was compared with the log-rank test. Univariate and multivariate models using proportional hazards regression were applied as an exploratory tool to assess the effect of the selected markers on DSS. Multivariate models were employed to adjust for possible confounding effect to classical clinicopathological features including tumor grade, tumor size, axillary lymph node status, HER2/*neu*, and estrogen receptor (ER)/ progesterone receptor (PR) status. The predictive power of the models was assessed as time dependent Area Under the Receiver Operatic Characteristic Curves (AUC(t)) and summarized by the concordance index (C-index) [17]. The C-index varies between 0.5 (no predictive power) and 1 (perfect prediction). Associations were evaluated by using the *χ*2-test or *t*-test, where appropriate. The probability (*P*) values were two-sided and considered statistically significant if *P* <0.05. All statistical manipulations were performed using the SPSS version 20.0 and R 2.14.0 statistical software.

## Results

### Clinicopathological characteristics

Eighty FFPE specimens from primary invasive breast tumors were initially collected for use in the present study. Due to loss of biopsy cores, insufficient tumor cells present in the cores or affluence of necrotic tissue, 72/80 FFPE specimens were evaluated for CCNB2, KIAA0101, SLC27A2, ASPM, and CDCA7 immunostaining. The mean age of the patients was 54.5 years with a range of 27–73 years. In total, 65% of the patients were over 50 years of age. Tumor size distribution was 20% for ≤ 2 cm, 70% for 2–5 cm, and 10% for > 5 cm. Among the patients, 35% were axillary lymph node-negative and 65% were axillary lymph node-positive. Lymph node-positive patients were further subdivided into two sub-groups based on the number of node metastases, 1–3 (46%) and ≥ 4 (54%). The tumors were stratified according to histological grade as defined by Bloom, Richardson, Elston/Ellis (BRE) grading system [8] identifying 51% of patients with grade I and II tumors, and 49% with grade III tumors. Thirty-nine percent of the patients (26/67 available fresh-frozen samples) were HER2/*neu* positive and 61% were HER2/*neu* negative at the DNA level. The cohort consisted of long-term survivors (46%, ≥8-year survival) and short-term survivors (54%, <8-year survival). Further detailed clinical information is presented in Table 1.

### Increased cytoplasmic expression of CCNB2 is associated with unfavorable prognosis

**Figure 1:**
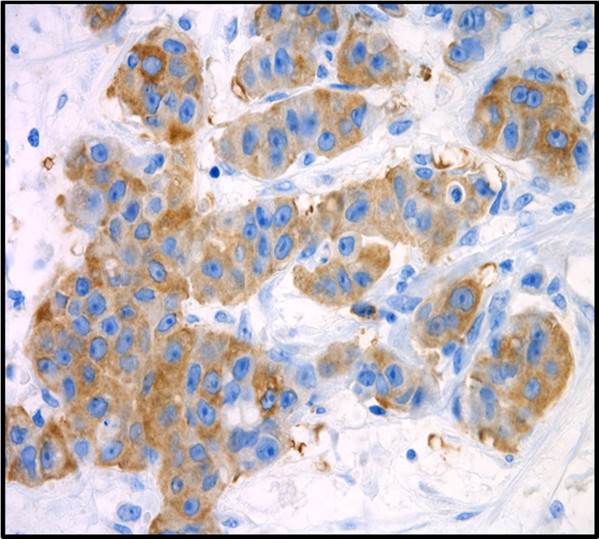
Immunohistochemical detection of CCNB2 expression in primary invasive breast tumors.

**Figure 2:**
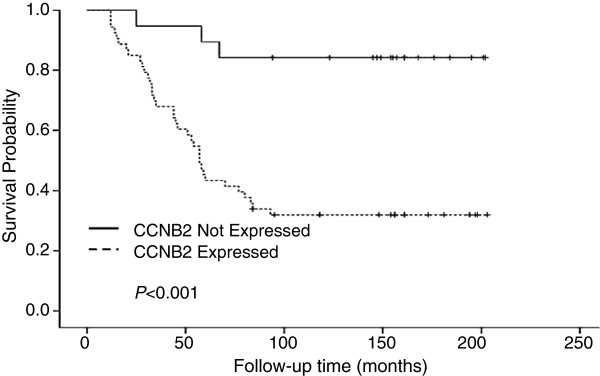
**Kaplan-Meier curves illustrating the effect of CCNB2 expression on disease specific survival in breast cancer.** Dashed line represents patients whose tumors over-expressed CCNB2 and solid line represents patients whose tumors did not. The *p*-values for the difference between the curves were calculated using log-rank test.

The cytoplasmic localization of the CCNB2 protein was detected in 74% of the samples, of which 92% of tumors from short-term survivors were CCNB2 positive (Figure 1). The univariate Cox proportional hazards regression analysis revealed that cytoplasmic CCNB2 expression was significantly associated with DSS (HR, 6.1; 95% CI: 2–20; Table 2). The effect of CCNB2 protein expression on patient DSS was evaluated by Kaplan-Meier analysis. As seen in Figure 2, over-expression of the CCNB2 protein had an adverse effect on survival rates (*P* = 0.001).

**Table 2:** Cytoplasmic CCNB2 staining, clinicopathological characteristics and univariate Cox Regression analysis in 80 invasive breast cancer patients

***Cytoplasmic CCNB2 expression***						
**Characteristics**	**Negative**	**Positive**	P*	**HR**	**95% CI**	***P *¤**
**Age (years)**			0.79	1.5	0.8-2.9	0.2
27-50	6 (24)	19 (76)				
>50	13 (28)	34 (72)				
**Histologic type**			0.04	0.8	0.4-1.5	0.5
Ductal	17 (29)	41 (71)				
Lobular	0 (0)	8 (100)				
Ductal + lobular	1(100)	0 (0)				
Other	0 (0)	4 (100)				
**Tumor size (mm)**			0.88	1.0	1.0-1.0	1.0
pT1 (0–20)	3 (25)	9 (75)				
pT2 (>20-50)	14 (27)	38 (73)				
pT3 (>50)	2 (25)	6 (75)				
**BRE grade**			0.99	1.1	0.8-1.5	0.5
I-II	10 (28)	26 (72)				
III	9 (25 )	27 (75)				
**Axillary lymph node status**			0.90	1.0	0.5-2.0	1.0
Negative	6 (25)	18 (75)				
Positive	13 (27)	35 (73)				
**No. of axillary lymph nodes**			0.34	1.0	0.7-1.4	0.8
1-3	4 (19)	17 (81)				
≥4	9 (33)	18 (67)				
**ER/PR status**			0.40	1.1	0.6-2.1	0.8
Negative	11 (32)	23 (68)				
Positive	8 (21)	30 (79)				
**HER2/*neu *status**			0.9	0.7	0.3-1.5	0.4
Positive	7 (32)	15 (68)				
Negative	10 (27)	27 (73)				
**ASPM**			0.03	0.9	0.5-20	0.9
Negative	10 (45)	12 (55)				
Positive	24 (37)	41 (63)				
**KIAA0101**			0.70	1.1	0.5-2.3	0.8
Negative	16 (48)	17 (52)				
Positive	3 (8)	36 (92)				
**SLC27A2**			0.08	0.6	0.3-1.3	0.2
Negative	11 (20)	43 (80)				
Positive	8 (44)	10 (56)				
**CCNB2**	19 (26)	53 (74)	0.004	6.1	2.0-20	0.003

*P*-value determined by chi-square test. * *P*-value of the correlation between CCNB-2 expression and clinicopatholigical parameters. ¤ *P*-value of hazard ratio (HR). BRE, Bloom, Richardson, Elston/Ellis; CI, confidence interval. All parameters were coded as 0 (negative) and 1 (positive) except as noted. Pathologic tumor size was coded as 1 (0-20mm), 2 (>20-50mm) and 3 (>50mm). Histologic type was coded as 1 (ductal), 2 (lobular ductal), 3 (ductal and lobular) and 4 (other). (N) = No. of patients (%).

**Figure 3:**
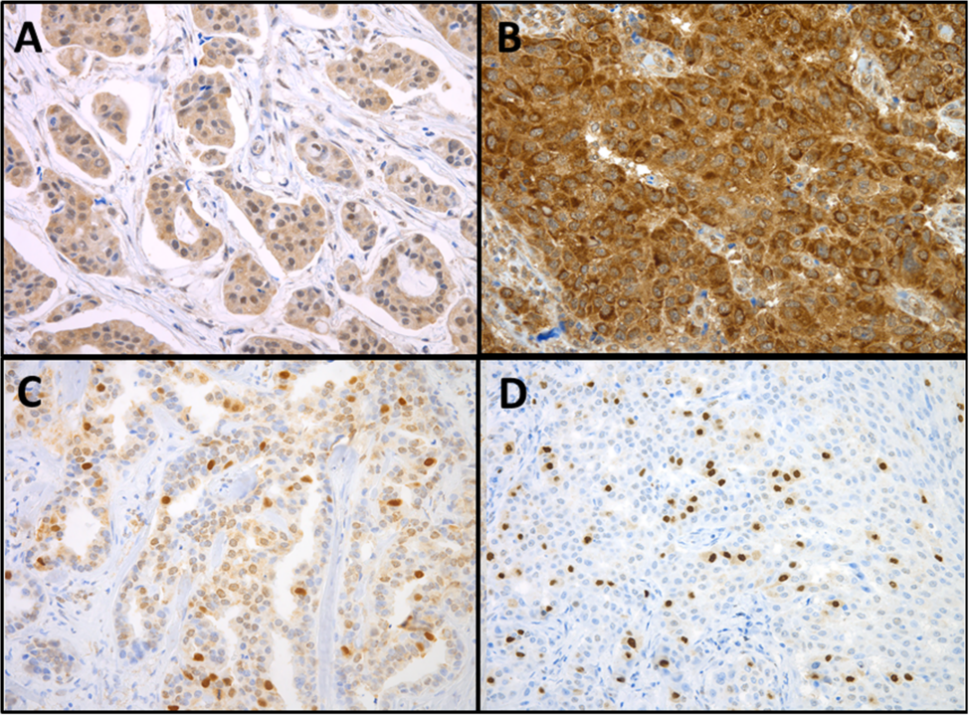
Immunohistochemical detection of ASPM (A), CDCA7 (B), KIAA0101(C) and SLC27A2 (D) protein levels in primary invasive breast tumors.

Approximately 69% of the 72 samples were positive for ASPM staining, displaying mainly granular nuclear staining. The expression of ASPM was similar regardless of the survival group. The CDCA7 protein was strongly expressed in the cell nucleus in all samples. The KIAA0101 protein was equally expressed in the cell nucleus in 79% of the analyzed specimens. Thirty percent of the long-term survivors and 20% of the short-term survivors expressed SLC27A2 in the cell cytoplasm (Figure 3). Furthermore, Kaplan-Meier analysis of CDCA7, ASPM, KIAA0101, and SLC27A2 were also performed. No differences in DSS in relation to protein expression were seen (data not shown).

### CCNB2 is an independent prognostic marker for breast cancer

**Figure 4:**
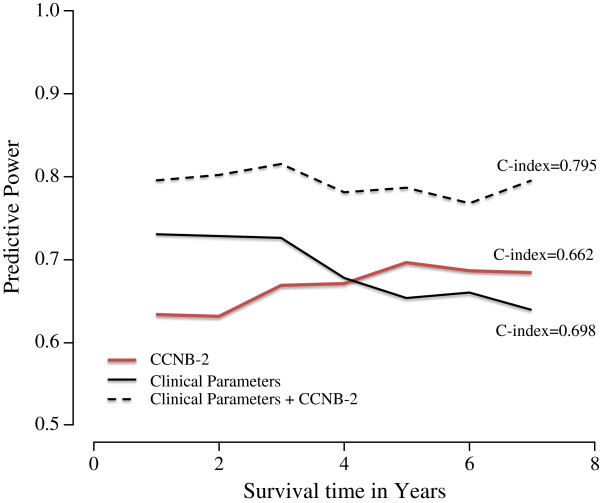
**Time dependent evolution of the prognostic models based on the CCNB2 expression only, clinical parameters only and finally a multivariate model containing both CCNB2 and the clinical parameters.** The time-dependent AUC values (AUC(t)) describes the predictive power of the models at different time-points.

For statistical analysis, the tumors were stratified into four groups according to CCNB2 protein levels and DSS status. As seen in Table 2, CCNB2 protein levels were significantly correlated with histological tumor type (*P =* 0.04), but no association of CCNB2 protein expression with the clinicopathological characteristics age, histological grade, nodal status, tumor size, ER/PR or HER2/*neu* status were identified. However, in the two survival groups a significant correlation between CCNB2 and ASPM protein expression was found (*P* = 0.03). Furthermore, histological grade, axillary lymph node status, tumor size, ER/PR status HER2/*neu* status, and cytoplasmic CCNB2 expression were included in multivariate survival analysis which showed that CCNB2 over-expression was an independent prognostic marker for disease specific survival in breast cancer (*P* = 0.003; 95% CI: 2–20; Table 3). As presented in Figure 4, we further calculated the C-index by using three models (CCNB2 with clinicopathological parameters and both models alone) to evaluate the predictive power of CCNB2. The C-index for CCNB2 alone was 0.662 with enhanced predictive accuracy over time. Furthermore, the predictive power for CCNB2 protein expression was higher together with the analyzed clinicopathological parameters (C-index = 0.795) than with the clinicopathological parameters alone (C-index = 0.698) for predicting breast cancer specific-survival within 8-years follow-up. The difference between the CCNB2 and clinicopathological models was slightly less convincing. In addition, we noted that the predictive accuracy for the clinicopathological model without CCNB2 displays a considerable decreasing tendency over time.

**Table 3:** Multivariate Cox regression analysis on disease-specific survival in 80 invasive breast cancer patients

**Disease specific survival**					
**Characteristics**	**β**	**SE**	**HR**	**95% CI**	*P*-value*
BRE grade	0.14	0.17	1.16	0.83-1.61	0.39
HER2/*neu* status	0.18	0.40	0.83	0.39-1.80	0.64
Axillary lymph node status	0.03	0.35	1.03	0.53-2.04	0.64
Pathologic tumor size	0.001	0.01	0.10	0.98-1.02	0.97
ER/PR status	0.03	0.35	0.99	0.49-1.95	0.94
CCNB2	1.82	0.61	6.14	1.87-20.1	0.003

* Determined by chi-square test.

β: Regression coefficient; SE: standard error of β; HR: hazard ratio; and CI: confidence interval. BRE: Bloom, Richardson, Elston/ Ellis; ER/PR: Estrogen/progesterone receptor. Coding of characteristics: BRE, HER2/*neu* status, Axillary lymph node status, ER/PR and CCNB2 were coded as 0 (negative) and 1 (positive) respectively. Pathologic tumor size was coded as 1 (0-20mm), 2 (>20-50mm), and 3 (>50mm).

### Quantitative real-time PCR analysis

**Figure 5:**
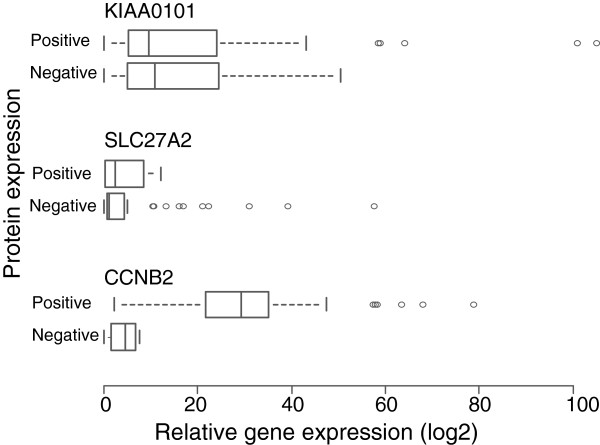
**The association of *CCNB2*, *KIAA0101*, and *SLC27A2 *mRNA levels to their corresponding protein levels in breast cancer patients.** The box plots Positive and Negative indicate corresponding gene expression levels for each protein. The mRNA expression of *CCNB2* was consistent with the IHC findings. There was no relationship between *KIAA0101* and *SLC27A2* mRNA with their corresponding protein expression.

Quantitative RT-PCR was performed to measure mRNA levels for the *CCNB2*, *KIAA0101*, *SLC27A2*, and HER2/*neu* genes and to validate the IHC and FISH results. We observed a positive association between the mRNA and protein levels of CCNB2 (*t*-test, *P<*0.001; Figure 5). The overall concordance between immunohistochemistry and qRT-PCR for CCNB2 was 94% (58/62). The four discordant cases showed CCNB2positivity, but low mRNA expression. A significant association between the gene amplification and mRNA levels of HER2/*neu* was also detected (*t*-test, *P* 0.005; data not shown). The relationship between the HER2/*neu* DNA copy number and mRNA expression was 94% (58/62). Three of the discordant samples revealed normal DNA copy numbers, but were highly expressed on the mRNA level. High *KIAA0101* and *SLC27A2* mRNA levels were detected in 92% and 83% of the samples, respectively. However, there was no relation between *KIAA0101* and *SLC27A2* mRNA levels and their corresponding protein levels (*t*-test, *P* = 0.776 and *P* = 0.973 respectively, Figure 5), indicating the expression of *KIAA0101* and *SLC27A2* mRNA levels appear to be independent of the presence of protein.

## Discussion

Despite the use of combination therapies including surgery and other systemic treatments (radiotherapy, endocrine therapy, and anticancer agents), many breast cancer patients will ultimately develop metastatic disease, which remains to be essentially incurable. Therefore, identification of novel prognostic and predictive biomarkers for breast tumors is needed and remains a long awaited priority to enhance individualized treatment. Recently, we identified molecular gene signatures associated with aggressive breast cancer [7]. Here, we present a validation of the prognostic role of five selected candidate biomarkers (CCNB2, ASPM, KIAA0101, CDCA7, and SLC27A2) included in these gene signatures using an independent breast cancer cohort.

In the present study, elevated CCNB2 protein levels were significantly associated with shorter DSS. Prominently, the HR of patients whose tumors were CCNB2-positive was 6.1 corresponding to a dramatic increase in probability of mortality compared with patients whose tumors had very little or no expression.

It is well-known that different cyclins regulate the eukaryotic cell cycle in association with cyclin dependant kinases (cdks) at specific points of the mitotic cycle. Cell cycle progression follows periodic alternations in the protein levels of cyclins, D, E, A and B. Deregulated levels of cyclins have been frequently observed in breast cancer [18]. Elevated levels of cyclin E was significantly correlated with DSS in patients with breast cancer [19]. Amplification and/or overexpression of cyclin D1 have been correlated to poor prognosis in breast cancer patients [20]. The Cyclin A upregulation was reported to be associated with increased risk of recurrence among breast cancer patients with node negative tumors [21]. The *CCNB2* gene is a member of the B-type cyclin family, including cyclin B1 and B2. It is involved in the G2-M transition in eukaryotes by activating CDC2 kinase and its inhibition induces cell cycle arrest [9,22,23]. In agreement with a crucial role in cell growth, numerous studies detected overexpression of CCNB2 in human tumors, including lung, colorectal adenocarcinoma, and pituitary adenomas [24-27]. Serum circulating *CCNB2* mRNA levels were found to be higher in lung and digestive tract cancer patients compared to normal controls and were correlated with cancer stage and metastasis status [28]. Furthermore, the *CCNB2* gene was included in the set of genes detected in node-negative breast tumors associated with poor prognosis [29]. Our results suggest that CCNB2 has an oncogenic potential and its overexpression may give some proliferative advantage. We have shown a significant association of CCNB2 protein expression with breast tumor type, indicating that the CCNB2 protein levels were unevenly distributed among the histological types. Notably, no statistically significant differences were identified between CCNB2 protein levels in relation to patient age, tumor size, tumor grade, ER/PR status, HER2/*neu*, stage, and axillary lymph node status. However, a dissimilar trend has been reported in serum circulating *CCNB2* mRNA, since it was found to correlate with both grade and metastasis status [28]. The multivariate analysis including CCNB2 and several clinicopathological parameters further verified that CCNB2 remained an independent prognostic indicator for DSS. These data indicate that tumors with histological grade (I, II and III), axillary lymph node status (positive, negative), tumor size (0–2, 2–5, and >5), ER/PR status (positive, negative) and HER2/*neu* status, exhibiting CCNB2 protein expression, have a more unfavorable prognosis, with an increased risk of overall shorter survival rates. Moreover, the predictive power of CCNB2 in addition to the clinicopathological parameters model was slightly higher compared to the lower C-index of the model including all clinicopathological parameters alone. Thus, the accuracy in patient prognosis may be improved by measuring CCNB2 expression in cases of breast cancer.

The ASPM protein was reported to participate in spindle organization, spindle orientation, mitotic progression, and cytokinesis [10,11,30,31]. Furthermore, the ASPM protein is over-expressed in various cancers [10,32-35] and its knockdown inhibits tumor proliferation [32]. There was no association between elevated ASPM protein levels and DSS or any other clinical parameters, suggesting that ASPM may be involved only in cancer initiation. A significant correlation between CCNB2 and ASPM was observed. The induction of ASPM and CCNB2 has previously been reported to regulate the G2/M cell cycle progression [36]. Thus, elevated levels of CCNB2 may reflect a functional correlation with ASPM overexpression, which could play a role in the progression of breast carcinoma. Furthermore, up-regulation of CCNB2 and ASPM was detected in glioblastoma multiforme xenograft tumors and *de novo* glioblastoma multiforme tumors [37]. Activation of *CCNB2* and *ASPM* genes induces tumorigenic phenotypes in a number of cancers, whereas their inhibition abrogates cellular proliferation in mice and induces genomic instability [23,38].

The *CDCA7* gene has been implicated in neoplastic transformation and it is one of the downstream targets of the *Myc* oncogene [13]. Interestingly, high nuclear expression of CDCA7 was seen in all the analyzed tumors in the present study. It is known that deregulation of cell cycle control is a fundamental feature of cancer pathogenesis, therefore it was not unexpected that CDCA7 protein has been observed to be expressed at high levels in almost all selected tumors. KIAA0101 is predominantly expressed in mitochondria and partially in nuclei, playing an essential role in the regulation of DNA repair, cell cycle progression, and cell proliferation [12]. Moreover, the *KIAA0101* gene is over-expressed in tumors of the esophagus [39], colon [40], lungs [41,42], and breast [43]. KIAA0101-positivity was observed in 79% of the immunostained tumors. Up-regulation of KIAA0101 was confirmed by real time qRT-PCR, which showed the over-expression of the gene at the mRNA level in 92% of the studied tumors. The possibility of tissue heterogeneity, accounting for the discordance between mRNA and protein expression cannot be excluded. In addition, there was no association between elevated CDCA7 and KIAA0101 protein levels and DSS or any other clinical parameters. The CDCA7, and KIAA0101 may therefore only have role in tumor initiation. Discordant results were detected between mRNA and protein expression of SLC27A2. High mRNA expression was detected in 83% of the analyzed tumors, but protein expression was only seen in 25%, possibly owing to posttranscriptional regulation and differences in mRNA, and protein turnover rates or poor specificity of the antibody used for IHC [44,45]. These findings suggest that down-regulation of SLC27A2 in the selected tissues at the protein level may contribute to disease progression. Indeed, this gene was reported to regulate the tumor suppressor gene PARP and decreased SLC27A2 expression levels were found in the metastatic compared to the non-metastatic neuroendocrine tumors [46]. However, no significant difference could be seen on the effect of SLC27A2 protein expression on DSS in breast cancer, nor could any association between the protein expression of SLC27A2 and the conventional clinical characteristics be observed.

To our knowledge, this is the first study in breast cancer patients reporting *CCNB2* as a prognostic marker for unfavorable patient prognosis. Since several publications revealed that altered CCNB2 expression is seen in many cancer types, therefore further investigation to elucidate the mechanism by which CCNB2 exerts its effects may prove useful in the development of novel anticancer agents.

## Conclusions

In summary, we report here that CCNB2 expression represents a threshold that can stratify breast cancer patients in a high risk group associated with an increased probability of mortality when compared to 8-year survivors. Moreover, our data suggests that CCNB2 is a potential independent prognostic factor and may be useful in conjunction with other clinicopathological features in breast cancer.

## Competing interests

The authors declare that they have no competing interests.

## Authors' contributions

ES and SH performed the immunohistochemistry. AK evaluated the immunostained breast cancer tissues. AK, ZE, and KG provided the clinical information. ES performed the fluorescence *in situ* hybridization and the qRTPCR. ES and SN performed the statistical analysis. ES interpreted the results. ES and KH wrote the paper. TP, SN, SH, AK, ZE, PK, and KH critically revised the manuscript. All authors read and approved the final manuscript.

## Pre-publication history

The pre-publication history for this paper can be accessed here:

http://www.biomedcentral.com/1471-2407/13/1/prepub
